# Prognostic Value of Oxidative Stress Markers in Patients with Pulmonary Arterial or Chronic Thromboembolic Pulmonary Hypertension

**DOI:** 10.1155/2019/3795320

**Published:** 2019-12-18

**Authors:** Anna Smukowska-Gorynia, Piotr Rzymski, Justyna Marcinkowska, Barbara Poniedziałek, Anna Komosa, Artur Cieslewicz, Sylwia Slawek-Szmyt, Magdalena Janus, Aleksander Araszkiewicz, Stanislaw Jankiewicz, Iga Tomaszewska-Krajniak, Tatiana Mularek-Kubzdela

**Affiliations:** ^1^1st Department of Cardiology, Poznan University of Medical Sciences, Poznan, Poland; ^2^Department of Environmental Medicine, Poznan University of Medical Sciences, Poznan, Poland; ^3^Department of Computer Science and Statistics, Poznan University of Medical Sciences, Poznan, Poland; ^4^Department of Clinical Pharmacology, Poznan University of Medical Sciences, Poznan, Poland

## Abstract

Oxidative stress is regarded to play a crucial role in the pathophysiology of pulmonary arterial hypertension (PAH) and inoperable chronic thromboembolic pulmonary hypertension (CTEPH). This study evaluated the prognostic value of serum oxidative stress markers (malondialdehyde (MDA), total antioxidant capacity (TAC), catalase activity (CAT), and superoxide activity (SOD)) in patients with PAH and CTEPH (*n* = 45). During 13 months of follow-up (median 9 months), clinical deterioration occurred in 14 patients (including 2 deaths). On the Cox regression analysis, MDA, TAC, and CAT were associated with clinical deterioration (*p* = 0.0068, HR = 1.42, 95% CI: 1.10-1.82; *p* = 0.0038, HR = 0.033, 95% CI: 0.0032-0.33; and *p* = 0.046, HR = 0.20, 95% CI: 0.04-0.98, respectively). There was no significant difference in SOD (*p* = 0.53, HR = 0.97, 95% CI: 0.87-1.08). The cut-off value derived from ROC curve analysis was 3.79 *μ*M (*p* = 0.0048, AUC = 0.76, 95% CI: 0.62-0.91) for MDA, 0.49 mM (*p* = 0.027, AUC = 0.71, 95% CI: 0.18-0.47) for TAC, and 1.34 U/L (*p* = 0.029, AUC = 0.71, 95% CI: 0.55-0.86) for CAT. MDA in the group with deterioration was higher (*p* = 0.0041), while TAC as well as CAT were lower (*p* = 0.027 and *p* = 0.028, respectively) when compared to stable patients. Survival without clinical deterioration was significantly longer in patients with lower MDA (*p* = 0.037, HR = 0.37, 95% CI: 0.12-1.14, log-rank), higher TAC (*p* = 0.0018, HR = 0.19, 95% CI: 0.06-0.60, log-rank), and higher CAT (*p* = 0.044, HR = 0.31 95% CI: 0.11-0.88, log-rank). Markers of oxidative stress such as MDA, TAC, and CAT were associated with adverse clinical outcomes in patients with PAH and inoperable or residual CTEPH.

## 1. Introduction

Pulmonary arterial hypertension (PAH) is a progressive disease characterized by the proliferation and vasoconstriction of pulmonary arterioles resulting in an increase of pulmonary pressure and pulmonary vascular resistance that might lead to right ventricle failure and eventually death [1]. Similarly, remodeling of distal pulmonary arteries in inoperable chronic thromboembolic pulmonary hypertension (CTEPH) results in gradual right ventricle deterioration. The etiology of PAH is complex and multifactorial including genetic factors, usage of toxic substances, connective tissue diseases, congenital heart diseases, and portopulmonary hypertension, while the etiology of CTEPH is thromboembolic. CTEPH develops when the clot does not resolve and transforms into fibrous tissue occluding the pulmonary artery. Characteristics of CTEPH include also dysfunction and remodeling of the pulmonary microvasculature similar to the arteriopathy of PAH. Pulmonary endarterectomy (PEA) is the gold-standard treatment for patients with CTEPH with changes localized in the proximal pulmonary artery, while for patients who are ineligible for surgery or have persistent pulmonary hypertension following PEA, percutaneous baloon pulmonary (BPA) angioplasty or targeted medical therapy can be administered [2, 3]. Nevertheless, despite the different etiologies of PAH and CTEPH, symptoms of the right ventricle failure as a result of precapillary pulmonary hypertension are common for both groups. The development of modern treatment including specific drugs in PAH and CTEPH, as well as a growing number of invasive balloon pulmonary angioplasties (BPA) in CTEPH whose effect is to decrease the overload of the right ventricle, improves outcomes [3–7]. However, it still remains under debate which clinical and laboratory parameters have the highest prognostic value in patients' risk stratification and determining the optimal time of therapy escalation remains a challenge for physicians. Among blood markers, only brain natriuretic peptide (BNP) and N-terminal-pronatriuretic peptide (NT-pro-BNP) are widely used validated prognostic biomarkers included in the multiparametric risk assessment approach according to the European Society of Cardiology guidelines [8].

Chronic upregulated inflammatory response has been widely acknowledged as a crucial pathogenic element of PAH and inoperable CTEPH [9, 10]. As shown using *in vitro* and *in vivo* experimental models and clinical data, oxidative stress, an imbalance between the generation of reactive oxygen species (ROS) and the biological system's ability to detoxify the reactive intermediates or to repair the resulting damage, appears as a significant mediator in the pathophysiology of PAH. Chronically increased ROS levels can uncouple endogenous nitric oxide (NO), which otherwise has a protective role, via the oxidation of its cofactors or direct oxidation which results in the production of superoxide (O_2_^•-^), further scavenged by NO to form peroxynitrite (ONOO^−^). Elevated levels of this product can in turn diminish endothelial nitric oxide synthase and prostacyclin synthase [11–15]. This subsequently leads to decreased vessel capacity to produce vasodilators such as NO and prostacyclin. Moreover, ROS and peroxides upregulate the COX expression, which eventually leads to the inactivation of prostacyclin synthase and an increase in thromboxane A2 [16, 17]. All in all, this supports endothelial dysfunction, vasoconstriction, and increased vascular tone [16, 18, 19]. Moreover, oxidative stress may directly or indirectly promote inflammation by the activation of stress kinases such as c-Jun activated kinase, extracellular signal-regulated kinase, p38, and redox-sensitive transcription factors, such as nuclear factor- (NF-) *κ*B and activator protein-1 [20]. Such induction of inflammation may further contribute to PAH progression and worsening of its outcome [21, 22]. The relation between oxidative stress in patients with PAH and inoperable CTEPH has been not fully explored. However, it has been preliminary shown that CTEPH patients may reveal increased serum malondialdehyde (MDA) while superoxide dismutase content may be decreased in PAH [23]. Patients with PAH have also been demonstrated to exhibit increased plasma MDA level [24]. The other study has shown that patients with chronic obstructive pulmonary disease combined with PAH displayed higher serum levels of reactive oxygen species and superoxide dismutase but surprisingly lower MDA concentration than patients with pure COPD [25]. Considering that oxidative stress may play an important role in the remodeling of the precapillary pulmonary arteries and associated events, it is of high interest to further explore whether its markers may have a prognostic value on disease progression and outcome in PAH and CTEPH.

The present study assessed the prognostic value of the following serum markers of oxidative stress in patients with PAH and inoperable or residual CTEPH: MDA concentration, total antioxidant capacity (TAC), and activities of superoxide dismutase (SOD) and catalase (CAT). All of these parameters are broadly employed to evaluate redox balance in various clinical conditions [26–29]. MDA is a hallmark of the peroxidation of lipids (one of the adverse outcomes of oxidative stress [30]); TAC reflects the overall balance between free radicals and low molecular weight, chain breaking antioxidants [31]; and SOD and CAT are the main elements of the enzymatic antioxidant system [32]. This was revealed for the first time when a follow-up (up to 13 months) was performed in patients with PAH and CTEPH after the level of oxidative stress markers was evaluated. The study contributes to the understanding of the prognostic role of selected biochemical markers in the outcome of these diseases.

## 2. Materials and Methods

### 2.1. Patients

Forty five patients (PAH, *n* = 28, and CTEPH, *n* = 17) were studied. The PAH group included patients with IPAH (54%, *n* = 15) and PAH associated with congenital heart disease (32%, *n* = 9) and connective tissue disease (14%, *n* = 4). Most patients had WHO FC II and were predominantly female. The CTEPH group included patients with inoperable lesions in pulmonary arteries (88%, *n* = 15) as well as patients after pulmonary artery thromboendarterectomy (PEA) with residual pulmonary hypertension after operation (12%, *n* = 2). Detailed baseline characteristics are shown in Table 1. Haemodynamic data and targeted treatment are shown in Table 2. The study group included approximately 80% of the population of patients in western Poland, treated with PAH- and CTEPH-specific drugs in 2017-2019 in the Poznan University of Medical Sciences.

The diagnosis of PAH and CTEPH was established on approved criteria [8] and confirmed by right heart catheterization. Operability of vascular lesions in CTEPH was assessed by a multiexpert team including a thoracic surgeon experienced in PEA and a cardiologist trained in BPA. When the vascular lesions in CTPEH were classified as inoperable, the BPA procedure was performed and treatment with riociguat was started simultaneously. In each patient, BPA was performed as a series of staged procedures. All BPAs were made via the right femoral vein approach under fluoroscopic control. At the beginning of the procedure, an intravenous bolus of unfractionated heparin (2000-5000 units) was administered, and then, heparin doses were repeated after each hour of the procedure. Typically, a MP, JR4, or JL4, 6F guiding catheter (Launcher; Meditronic, Minneapolis, MN, USA) was introduced into the right or left pulmonary artery with the use of a 90 cm 6F vascular sheath (Flexor; Cook, Bloomington, IN, USA) to achieve a selective approach to the target vessel. Then, 0.014-inch guidewires (Whisper MS; Abbott Vascular, Santa Clara, CA, USA; Sion Blue, Asahi, Japan) were passed through the lesions and placed distally in the subsegmental artery. Subsequently, the target branches were dilated with multiple balloon inflations using semicompliant balloon catheters with a size between 1.25 and 10 mm. The balloon parameters were adjusted to the type of lesion assessed with angiography. The next steps include contrast injection into the treated vessel and the evaluation of the angiographic effect of the procedure. A series of BPA was terminated when the mPAP was lower than 25 mmHg or when there were no treatable lesions in angiographic assessment. Furthermore, all individuals underwent medical assessment, performed in a serial fashion, every 3-6 months: medical interview with determination of the WHO functional class (FC), physical examination, blood sample collection for routine analysis, transthoracic echocardiography, 6 min walk distance (6MWD), and cardiopulmonary exercise test (CPET). The end point of clinical worsening comprised of WHO FC change, the need for escalation of therapy, unscheduled hospitalization due to disease progression, or death.

### 2.2. Serum Collection

Peripheral venous blood samples of studied patients were collected after overnight fasting. Following centrifugation at 1000 × g for 10 min, the serum was collected and stored at −80°C. The study was approved by the Local Bioethical Committee of the Poznan University of Medical Sciences in Poland (approval no. 531/17; date of approval: 11th May 2017). All patients signed a written consent form. The total antioxidant capacity (TAC), malondialdehyde (MDA) content, and activity of catalase (CAT) and superoxide dismutase (SOD) were analyzed in the collected serum.

### 2.3. Total Antioxidant Capacity

TAC was assessed by a method adapted from Rice-Evans and Miller [33] which is based on the antioxidant-induced inhibition of the absorbance of the radical cation of 2,2′-azino-bis (3-ethylbenzothiazoline 6-sulphonate) (ABTS). The ABTS radical cation is formed by the interaction of 150 *μ*M ABTS with the ferrylmyoglobin radical species, generated by the activation of 2.5 *μ*M metmyoglobin with 75 *μ*M H_2_O_2_. Antioxidant agents suppress the formation of the ABTS radical cation. The assay was performed with the use of the Antioxidant Assay Kit (Sigma-Aldrich, USA). Briefly, ABST, myoglobin, and 10 *μ*L of serum were mixed, and the reaction was initiated by the addition of H_2_O_2_. Following the incubation for 5 min at 21°C, the absorbance of the product was read at 735 nm and compared to a calibration curve prepared using Trolox (6-hydroxy-2,5,7,8-tetramethylchroman-2-carboxylic acid), a water soluble analogue of vitamin E commonly used in antioxidant assays as a standard antioxidant [31]) (0.0–2.0 mM; *r*^2^ = 0.98) and given as mM Trolox equivalents. Three technical replicates were performed for each serum sample.

### 2.4. Malondialdehyde Concentration

Lipid peroxidation was evaluated by means of serum levels of thiobarbituric acid-reactive substances (TBARs) that include malondialdehyde (MDA), one of the most commonly reported biomarkers of lipid peroxidation in clinical studies [26]. Here, the MDA concentration was assessed using a TBARS Assay Kit provided by Cayman Chemical (Ann Arbor, MI, USA). Thawed serum samples (100 *μ*L) were supplemented with butylated hydroxytoluene to prevent artificial lipid peroxidation. The samples were then centrifuged (1600 × g, 10 min, 4°C) to remove insoluble material, and 100 *μ*L of supernatants were transferred to a microcentrifuge tube and supplemented with 800 *μ*L of thiobarbituric acid (TBA) to generate an MDA-TBA adduct. To accelerate the process, samples were incubated at 95°C for 60 min, placed on an ice bath for 10 min to inhibit the reaction, and centrifuged (1600 × g, 10 min, 4°C). The final product was measured colorimetrically at 532 nm), and the absorbance values were compared to a calibration curve prepared using the MDA standard (0.0–50.0 *μ*M; *r*^2^ = 0.99) (Cayman Chemical, USA). Three technical replicates were performed for each serum sample.

### 2.5. Superoxide Dismutase Activity

SOD (EC 1.15.1.1.) activity in serum was measured using the SOD Determination Kit (Sigma-Aldrich, St. Louis, MO, USA). Briefly, in this assay, a superoxide radical, generated from the conversion of xanthine to uric acid and H_2_O_2_ by xanthine oxidase, converts the tetrazolium salt WST-1 to water-soluble WST-1 formazan. The SOD reduces concentrations of the superoxide anion radical and thereby lowers the rate of formation of WST-1 formazan. The absorbance of this final product was measured at 450 nm, and SOD activity was calculated as a percent of inhibition of WST-1 formazan formation.

### 2.6. Catalase Activity

Catalase (CAT, EC 1.11.1.6) activity in serum was assayed with the Catalase Assay Activity Kit (Abcam, Cambridge, UK). Briefly, in this assay, CAT reacts with H_2_O_2_ to produce water and oxygen while its unconverted pool reacts with the OxiRed™ Probe to produce a product which is measured at 570 nm. For each sample, a High Control in which the CAT activity was inhibited at the beginning of the procedure, was prepared. The final amount of degraded H_2_O_2_ was calculated by subtracting the absorbance measured and a sample from that measured in the High Control. These values were compared to a calibration curve (*r*^2^ = 0.99) prepared using an H_2_O_2_ standard (Abcam, Cambridge, UK).

### 2.7. Statistical Analyses

The distribution of all variables was verified with the Shapiro-Wilk test for normality. Data is shown as mean ± SD or median values with interquartile range (IQR) as appropriate. We compared group with deterioration vs. stable group using the Fisher exact test for categorical data and the Mann-Whitney *U* test for continuous data. We assessed the impact of the level of MDA, TAC, and CAT as well as specific therapy on clinical deterioration by creating a Cox proportional hazard model. Receiver operating characteristic (ROC) curve was used to determine a cut-off point associated with a higher probability of clinical deterioration. A survival curve was plotted as estimated by the Kaplan-Meier method and compared with others using the log-rank test. *p* values < 0.05 were considered statistically significant. Statistical analysis was performed using the PQStat version 1.6.8 and Statistica version 12.

## 3. Results

**Figure 1:**
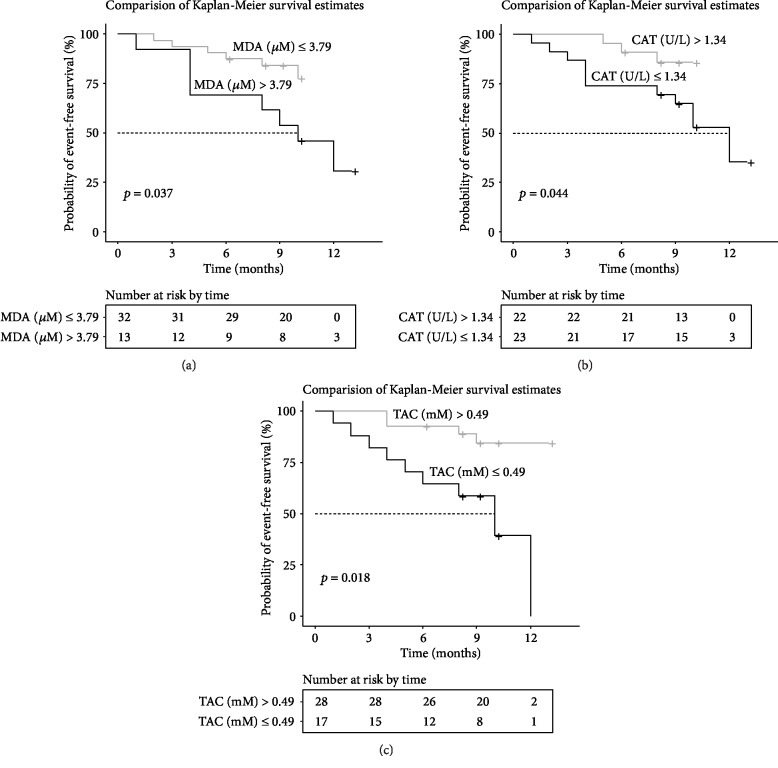
(a) Kaplan-Meier survival curves demonstrate survival estimates in patients with pulmonary arterial or chronic thromboembolic hypertension, graded by malondialdehyde (MDA) levels below and above receiver operating characteristic-derived values. (b) Kaplan-Meier survival curves showing survival estimates in patients with pulmonary arterial or chronic thromboembolic hypertension, graded by total antioxidant capacity (TAC) levels below and above receiver operating characteristic-derived values. (c) Kaplan-Meier survival curves showing survival estimates in patients with pulmonary arterial or chronic thromboembolic hypertension, graded by catalase activity (CAT) levels below and above receiver operating characteristic-derived value.

During 13 months of follow-up (median 9 months), clinical deterioration occurred in 14 patients (including 2 deaths). On the Cox regression analysis, MDA, TAC, and CAT were associated with clinical deterioration (*p* = 0.0068, HR = 1.42, 95% CI: 1.10-1.82; *p* = 0.0038, HR = 0.033, 95% CI: 0.0032-0.33; and *p* = 0.046, HR = 0.20, 95% CI: 0.04-0.98, respectively). There was no significant difference in SOD (*p* = 0.53, HR = 0.97, 95% CI: 0.87-1.08). The cut-off value derived from ROC curve analysis was 3.79 *μ*M (*p* = 0.0048, AUC = 0.76, 95% CI: 0.62-0.91) for MDA, 0.49 mM (*p* = 0.027, AUC = 0.71, 95% CI: 0.18-0.47) for TAC, and 1.34 U/L (*p* = 0.029, AUC = 0.71, 95% CI: 0.55-0.86) for CAT. Survival without clinical deterioration was significantly longer in patients with lower MDA (*p* = 0.037, HR = 0.37, 95% CI: 0.12-1.14, log-rank, Figure 1(a)), higher CAT (*p* = 0.044, HR = 0.31, 95% CI: 0.11-0.88, log-rank, Figure 1(b)), and higher TAC (*p* = 0.0018, HR = 0.19, 95% CI: 0.06-0.60, log-rank, Figure 1(c)). There was no difference in serum TAC, MDA, CAT, and SOD between PAH and CTEPH groups. The observed levels of studied parameters are presented in Table 3.

Prostacycline treatment was associated with worse prognosis on the Cox regression analysis (*p* = 0.004, HR = 5.38, 95% CI: 1.71-16.95). There was, however, no difference in TAC, MDA, CAT, and SOD between patients receiving prostacyclines and those without their use (Table 4).

Patients with clinical deterioration were significantly more frequent in WHO FC III or IV (*p* = 0.0.17), had lower peakVO_2_ derived from pulmonary exercise test (*p* = 0.039), had more frequent fluid in the pericardium (*p* = 0.023), and had higher concentrations of troponin I (*p* = 0.017) and MDA (*p* = 0.0041), while activities of TAC (*p* = 0.027) and CAT (*p* = 0.028) were significantly lower compared to stable patients (Table 5).

## 4. Discussion

There is a large body of evidence that increased oxidative stress contributes to PAH pathogenesis. Elevated levels of urinary F2-isoprostanes, a biomarker of lipid peroxidation, was documented in patients with PAH and correlated inversely with vasoreactivity [34]. Moreover, F2-isoprostanes decreased in urine after epoprostenol treatment and correlated with haemodynamic and clinical improvement [35]. Similarly, vardenafil reduced the levels of oxidative stress biomarkers (such as 8-iso-prostaglandin-F2*α* and 3-nitrotyrosine in PAH patients), significantly increased endothelial NO synthase expression and superoxide dismutase activity, and downregulated nicotinamide adenine dinucleotide phosphate oxidase expression in rat lung tissue [36]. Oxidized guanosine was also elevated in the lungs of patients with PAH [37].

Recently, we have documented that neopterin concentration which increases the cytotoxic potential of activated macrophages and dendritic cells through promoting oxidative stress was elevated compared to the control group and predicted adverse outcomes in patients with PAH and inoperable CTEPH [38]. The results of another study show that SOD and glutathione peroxidase (GPx) activity are reduced in IPAH lungs, compared to healthy controls [39]. In the study of Irodova et al., MDA level was increase in PAH patients compared to healthy controls and was most pronounced in patients with severe pulmonary hypertension, while the results of glutathione peroxidase activity were inconclusive [24]. In animal models, in mutant bone morphogenetic protein receptor 2 mice, hyperoxia contributed to disease exacerbation [40].

An increasing number of studies focus on the evaluation of oxidative stress markers in various human diseases and treatment outcomes [24, 41]. For example, their value has been shown in predicting neurological outcome in postsurgical aneurysmal subarachnoid hemorrhage patients [42], mortality in hemodialysis patients [43], risk of complications in type 2 diabetes [44], and prognosis in colorectal and lymphocytic leukaemia [45, 46]. Their potential prognostic value in the clinical outcome of cardiovascular diseases (CVD) has also been highlighted with MDA, SOD, and CAT being one of the most promising biomarkers in this regard [47]. However, their use must be specified for particular clinical conditions.

In the present study, a set of four oxidative stress biomarkers in serum has been taken into account: CAT and SOD activity, MDA concentration, and TAC. The former two represent the core of an enzymatic antioxidant system: the general function of SODs is to catalyze the conversion of superoxide radical to H_2_O_2_, while CAT further converts it into water. As shown *in vivo*, SOD and CAT mimetics can reverse pulmonary vascular remodeling and PAH [48]. It is thus plausible that the activities of these enzymes may have a prognostic value in patients with PAH and CTEPH. The present study confirmed such value only in the case of CAT, which has one of the highest catalytic activities reported, close to the diffusion-controlled limit, thus playing the main role in the detoxification of H_2_O_2_. No effect was identified for SOD which is an interesting finding if one considers that patients with chronic obstructive pulmonary disease (COPD) combined with PAH were previously demonstrated to reveal significantly higher serum SOD levels than subjects with pure COPD [23]. However, it should be noted that various studies in patients with clinical conditions different from that of PAH have already produced contradictory results regarding CAT and SOD values [49]. This may result from a complex nature of interactions between these enzymes—as demonstrated, the accumulation of high contents of superoxide anion can additionally decrease CAT activity, whereas the increased H_2_O_2_ resulting from CAT inhibition can finally suppress SOD activity [50, 51]. Last but not the least, it should also be stressed that the measurement of activity of antioxidant enzymes may not be sufficient enough to unambiguously recognize whether tissues cope with ROS and oxidative stress or not; e.g., their high or low activities may represent a homeostatic redox state, a response to increased ROS level, or might as well be a result of the prolonged, harmful effect of oxidative stress [36].

The prognostic value was also demonstrated for the serum concentration of MDA, which along with 4-hydroxy-2-nonenal, is a major genotoxic product of lipid peroxidation [30]. This is unsurprising given the fact that peroxidation of lipids is known to be increased in various CVD, including PAH [26]. It has also been shown that MDA can have a prognostic value for survival in various groups of patients [52, 53]. An additional advantage of using MDA as a potentially prognostic marker of clinical outcome is that the performance of assays based on TBARS is relatively not difficult and not highly time-consuming, and a number of assay kits are commercially available. Investigating TAC is even less time-consuming and can provide a global information on the availability of enzymatic and nonenzymatic antioxidants, and free radicals and products of their reactions. In contrast, measuring specific antioxidant assays are expensive and cumbersome in performance and can only provide limited data on the assessed antioxidants [31, 54]. Importantly, in patients studied in the present research, TAC displayed the highest prognostic value. It is plausible that patients with PAH and inoperable CTEPH are undergoing chronic oxidative stress manifested on various molecular and biochemical levels, and the evaluation of TAC under such conditions may provide a more generalized but at the same time more accurate information on how redox imbalance may affect disease outcome and patient survival.

In the study group, 14 patients were treated with prostacyclines (treprostinil or iloprost). This may in turn potentially affect a systemic redox imbalance as these pharmaceuticals are regarded to reduce oxidative stress [35, 37]. However, the present study found no difference in TAC, MDA, CAT, nor SOD in patients receiving prostacyclines compared to patients treated with specific drugs acting through other pathways. On the other hand, according to the ESC guidelines, parental prostacyclines are recommended in more advanced disease (WHO FC III or IV) [8], which may explain the results of oxidative stress biomarkers with respect to targeted drug therapy. Indeed, in the study group, prostacycline treatment was associated with a higher probability of clinical worsening which may imply that in a nonselected population of patients, the potential positive effect of prostacyclines is diminished by disease severity.

As shown in Table 3, among the validated widely used prognostic clinical factors, WHO FC and peakVO_2_ derived from pulmonary exercise test and fluid in the pericardium significantly distinguished stable patients from patients with clinical worsening. These results suggest that a pulmonary exercise test has a higher value than 6MWT in disease prognostication. Interestingly, among the laboratory parameters, higher concentrations of troponin I (laboratory normal limits 0.056 ng/mL) and MDA and lower activities of TAC as well as CAT were observed in patients with clinical deterioration compared to stable patients, which indicate that studied oxidative stress biomarkers and troponin I may precede the change in NT-pro-BNP and measured simultaneously can be a better prognostic tool than NT-pro-BNP alone.

Although the study provided new insights into the prognostic value of oxidative stress markers in PAH and CTEPH, one should acknowledge its limitations. Firstly, this research encompassed a small cohort size and the observations would require confirmation from a larger population. Therefore, the reported findings should be considered as preliminary. Secondly, due to the small group of studied patients, we were unable to perform multivariable analysis. Although the selected markers and complimentary markers of oxidative stress were selected, there are number of other relevant antioxidant agents (e.g., glutathione and metal-binding proteins [55, 56]) or parameters related to oxidative damage (e.g., protein carbonylation [57]) that could also be potentially associated with patient outcome. Moreover, as long as the measurement of TAC, MDA, CAT, and SOD in serum provides an overview of systemic redox balance, it provides no real mechanistic insight into their role in PAH and CTEPH. Last but not least, the findings of the present study should rather be treated as of associative nature, not truly predictive, since the oxidative markers were not measured in sequential fashion or compared to baseline prior treatment.

## 5. Conclusions

In summary, serum markers of oxidative stress such as MDA concentration and TAC and CAT activities were associated with adverse clinical outcomes in patients with PAH and inoperable or residual CTEPH. MDA and TAC appeared to have the superior prognostic value.

## Figures and Tables

**Table 1:** Demographic and clinical characteristics of studied patients.

Characteristics	PAH (*n* = 28)	CTEPH (*n* = 17)
Gender (*n*, %)		
Female	20 (71)	10 (59)
Male	8 (19)	7 (41)
Age (years)	57 (36.5-68.5)	68 (62-73)
PAH etiology (*n*, %)		
IPAH	15 (54)	
PAH CHD	9 (32)	
PAH CTD	4 (14)	
WHO FC (*n*, %)		
I	5 (18)	1 (9)
II	14 (50)	11 (65)
III	9 (32)	4 (17)
IV	0 (0)	1 (9)
peakVO_2_ (mL/kg/min)	15.35 (12.0-20.3)	17.7 (13.9-19.9)
6MWT (m)	480 (350-563)	390 (240-503)
Right atrium area (cm^2^)	20 (16.0-31.5)	21 (19-27)
Fluid in pericardium (*n*, %)	3 (11)	4 (24)
NT-pro-BNP (pg/mL)	259.0 (111.0-718.0)	258 (168-476)
RDW (%)	13.9 (13.3-15.8)	14.9 (14.4-16.1)
Troponin I	0.0005 (0.0000-0.0390)	0.0000 (0.0000-0.0005)

IPAH: idiopathic pulmonary arterial hypertension; PAH CHD: pulmonary arterial hypertension associated with congestive heart defect; PAH CTD: pulmonary arterial hypertension associated with connective tissue disease; CTEPH: chronic thromboembolic pulmonary hypertension; WHO FC: World Health Organization Functional Class; peakVO_2_: peak oxygen consumption; 6MWT: 6-minute walking test; NT-pro-BNP: N-terminal B-type natriuretic peptide; RDW: red blood cell distribution width.

**Table 2:** Haemodynamic data and specific treatment.

Parameter	PAH (*n* = 28)	CTEPH (*n* = 17)
Haemodynamic parameters, median, IQR		
mPAP (mmHg)	44.5 (35.5-52.5)	36.0 (29.0-41.0)
PVR (Wood units)	469 (354.0-652.7)	264 (218.3-419.6)
CI (L/min/m^2^)	3.3 (2.96-4.05)	3.52 (3.13-4.07)
mRAP (mmHg)	6.4 (4.5-7.0)	5.0 (4.0-8.0)
SatO_2_mix (%)	72.3 (66.076.8)	75.8 (70.8-79.4)
PAH and CTEPH specific treatment		
Monotherapy (*n*, %)		
Riociguat	1 (4)	16 (94)
Sildenafil	4 (14)	0 (0)
Bosentan	4 (14)	0 (0)
Macicentan	1 (4)	0 (0)
Combined therapy (*n*, %)		
Sildenafil+treprostinil sc	6 (21)	0 (0)
Sildenafil+macicentan	3 (11)	0 (0)
Sildenafil+iloprost	2 (7)	0 (0)
Sildenafil+bosentan	1 (4)	0 (0)
Sildenafil+iloprost+bosentan	4 (14)	0 (0)
Sildenafil+oral treprostinil+macicentan	1 (4)	0 (0)
Sildenafil+oral treprostinil	1 (4)	
Baloon pulmonary angioplasty	0 (0)	15 (88)

mPAP: mean pulmonary artery pressure; PVR: pulmonary vascular resistance; CI: cardiac index; mRAP: mean right atrium pressure; SatO_2_mix: mixed venous oxygen saturation; PAH: pulmonary arterial hypertension; CTEPH: chronic thromboembolic pulmonary hypertension.

**Table 3:** Median (IQR) values of studied parameters of oxidative stress in PAH and CTEPH patients.

	PAH (*n* = 28)	CTEPH (*n* = 17)	*p* value
TAC (mM)	0.52 (0.36-0.71)	0.64 (0.53-0.73)	0.32
MDA (*μ*M)	2.91 (1.75-4.00)	2.63 (0.89-3.93)	0.59
CAT (U/L)	1.33 (1.16-1.70)	1.68 (1.22-2.56)	0.16
SOD (%)	79.16 (76.14-82.57)	79.89 (77.23-85.13)	0.34

PAH: pulmonary arterial hypertension; CTEPH: chronic thromboembolic pulmonary hypertension; TAC: total antioxidant capacity; MDA: malondialdehyde; CAT: catalase activity; SOD: superoxide activity; *p* value refers to Mann-Whitney *U* test.

**Table 4:** Median (IQR) values of studied parameters of oxidative stress in patients receiving or not prostacycline treatment.

	Patients with PC (*n* = 14)	Patients without PC (*n* = 31)	*p* value
TAC (mM)	0.48 (0.38-0.65)	0.64 (0.42-0.76)	0.15
MDA (*μ*M)	2.19 (1.63-3.35)	3.32 (1.30-4.02)	0.47
CAT (U/L)	1.46 (1.16-1.90)	1.29 (1.19-2.37)	0.85
SOD (%)	79.16 (74.79-82.48)	79.89 (77.23-84.80)	0.38

TAC: total antioxidant capacity; MDA: malondialdehyde; CAT: catalase activity; SOD: superoxide activity; PC: prostacyclines; *p* value refers to Mann-Whitney *U* test.

**Table 5:** Comparison of clinical and laboratory parameters in patients with clinical worsening vs. stable patients.

Characteristics	Clinical worsening (*n* = 14)	Stable (*n* = 31)	*p* value
Gender			0.17
Female	7 (50)	23 (74)	
Male	7 (50)	8 (26)	
Age (years)	64 (42-74)	62 (49-71)	0.64
WHO FC (*n*, %)			0.017
I or II	6 (43)	25 (81)	
III or IV	8 (57)	6 (29)	
peakVO_2_ (mL/kg/min)	14.2 (12.0-15.3)	17.3 (13.75-21.05)	0.039
6MWT (m)	380.5 (180-510)	480 (360-525)	0.17
RAA (cm^2^)	25.5 (19.0-37.0)	20.3 (16.0-26.9)	0.08
Fluid in pericardium (*n*, %)	5 (36)	2 (6)	0.023
NT-pro-BNP (pg/mL)	612.0 (98.0-1905.0)	206.0 (122.0-476.0)	0.13
RDW (%)	15.6 (13.8-16.2)	14.2 (13.3-15.0)	0.16
Troponin I (ng/mL)	0.042 (0.0010-0.115)	0.000 (0.0000-0.0005)	0.0017
mPAP (mmHg)	39.0 (34.0-48.0)	38.5 (30.0-50.0)	0.84
PVR (Wood units)	449.0 (290.0-592.0)	360.8 (247.0-536.0)	0.65
CI (L/min/m^2^)	3.4 (3.1-4.1)	3.5 (3.0-4.1)	0.97
mRAP (mmHg)	7.0 (4.0-8.0)	6.0 (4.0-7.0)	0.53
SatO_2_mix (%)	73.6 (66.0-75.8)	74.5 (68.1-79.4)	0.26
TAC (mM)	0.41 (0.24-0.48)	0.64 (0.54-0.72)	0.027
MDA (*μ*M)	3.86 (3.06-4.56)	2.25 (0.98-3.54)	0.0041
CAT (U/L)	1.23 (1.16-1.33)	1.57 (1.2-2.5)	0.028
SOD (%)	78.88 (75.40-83.61)	79.88 (77.02-82.77)	0.67

WHO FC: World Health Organization Functional Class; peakVO_2_: peak oxygen consumption; 6MWT: 6-minute walking test; RAA: right atrium area; NT-pro-BNP: N-terminal B-type natriuretic peptide; RDW: red blood cell distribution width; mPAP: mean pulmonary artery pressure; PVR: pulmonary vascular resistance; CI: cardiac index; mRAP: mean right atrium pressure; SatO_2_mix: mixed venous oxygen saturation; PAH: pulmonary arterial hypertension; CTEPH: chronic thromboembolic pulmonary hypertension; TAC: total antioxidant capacity; MDA: malondialdehyde; CAT: catalase activity; SOD: superoxide activity; *p* value refers to the Mann-Whitney *U* test or the Fisher exact test.

## Data Availability

The data used to support the findings of this study are available from the corresponding authors upon request.

## References

[1] Lai Y. C., Potoka K. C., Champion H. C., Mora A. L., Gladwin M. T. (2014). Pulmonary arterial Hypertension. *Circulation Research*.

[2] Kim N. H., Delcroix M., Jais X. (2019). Chronic thromboembolic pulmonary hypertension. *The European Respiratory Journal*.

[3] Ogawa A., Satoh T., Fukuda T. (2017). Balloon pulmonary angioplasty for chronic thromboembolic pulmonary Hypertension. *Circulation Cardiovascular Quality and Outcomes*.

[4] Torbicki A., Bacchi M., Delcroix M. (2019). Integrating data from randomized controlled trials and observational studies to assess survival in rare diseases. *Circulation. Cardiovascular Quality and Outcomes*.

[5] Sitbon O., Gomberg-Maitland M., Granton J. (2019). Clinical trial design and new therapies for pulmonary arterial hypertension. *European Respiratory Journal*.

[6] van Thor M. C. J., ten Klooster L., Snijder R. J., Post M. C., Mager J. J. (2019). Long-term clinical value and outcome of riociguat in chronic thromboembolic pulmonary hypertension. *IJC Heart & Vasculature*.

[7] Barnes H., Yeoh H. L., Fothergill T. (2019). Prostacyclin for pulmonary arterial hypertension. *Cochrane Database of Systematic Reviews*.

[8] Nazzareno Galiè M. H., Vachiery J. L., Gibbs S., Lang I., Torbicki A., Simonneau G. (2015). ESC/ERS Guidelines for the diagnosis and treatment of pulmonary hypertension. *The European Respiratory Journal*.

[9] Rabinovitch M., Guignabert C., Humbert M., Nicolls M. R. (2014). Inflammation and immunity in the pathogenesis of pulmonary arterial hypertension. *Circulation Research*.

[10] Quarck R., Wynants M., Verbeken E., Meyns B., Delcroix M. (2015). Contribution of inflammation and impaired angiogenesis to the pathobiology of chronic thromboembolic pulmonary hypertension. *The European Respiratory Journal*.

[11] Lin K. T., Xue J. Y., Sun F. F., Wong P. Y. (1997). Reactive oxygen species participate in peroxynitrite induced apoptosis in HL 60 cells. *Biochemical and Biophysical Research Communications*.

[12] Hink U., Oelze M., Kolb P. (2003). Role for peroxynitrite in the inhibition of prostacyclin synthase in nitrate tolerance. *Journal of the American College of Cardiology*.

[13] Cassuto J., Dou H., Czikora I. (2014). Peroxynitrite disrupts endothelial caveolae leading to eNOS uncoupling and diminished flow-mediated dilation in coronary arterioles of diabetic patients. *Diabetes*.

[14] Zou M., Martin C., Ullrich V. (1997). Tyrosine nitration as a mechanism of selective inactivation of prostacyclin synthase by peroxynitrite. *Biological Chemistry*.

[15] Nash K. M., Schiefer I. T., Shah Z. A. (2018). Development of a reactive oxygen species-sensitive nitric oxide synthase inhibitor for the treatment of ischemic stroke. *Free Radical Biology & Medicine*.

[16] Hardy P., Dumont I., Bhattacharya M. (2000). Oxidants, nitric oxide and prostanoids in the developing ocular vasculature: a basis for ischemic retinopathy. *Cardiovascular Research*.

[17] Majed B. H., Khalil R. A. (2012). Molecular mechanisms regulating the vascular prostacyclin pathways and their adaptation during pregnancy and in the newborn. *Pharmacological Reviews*.

[18] Sartori C., Allemann Y., Scherrer U. (2007). Pathogenesis of pulmonary edema: learning from high-altitude pulmonary edema. *Respiratory Physiology & Neurobiology*.

[19] Panieri E., Santoro M. M. (2015). ROS signaling and redox biology in endothelial cells. *Cellular and Molecular Life Sciences*.

[20] Rahman I., Adcock I. M. (2006). Oxidative stress and redox regulation of lung inflammation in COPD. *The European Respiratory Journal*.

[21] Price L. C., Wort S. J., Perros F. (2012). Inflammation in pulmonary arterial hypertension. *Chest*.

[22] Boukhenouna S., Wilson M. A., Bahmed K., Kosmider B. (2018). Reactive oxygen species in chronic obstructive pulmonary disease. *Oxidative Medicine and Cellular Longevity*.

[23] Zhang S., Yang T., Xu X. (2015). Oxidative stress and nitric oxide signaling related biomarkers in patients with pulmonary hypertension: a case control study. *BMC Pulmonary Medicine*.

[24] Irodova N. L., Lankin V. Z., Konovalova G. K., Kochetov A. G., Chazova I. E. (2002). Oxidative stress in patients with primary pulmonary hypertension. *Bulletin of Experimental Biology and Medicine*.

[25] Zhuan B., Yu Y., Yang Z., Zhao X., Li P. (2017). Mechanisms of oxidative stress effects of the NADPH oxidase-ROS-NF-*κ*B transduction pathway and VPO1 on patients with chronic obstructive pulmonary disease combined with pulmonary hypertension. *European Review for Medical and Pharmacological Sciences*.

[26] Komosa A., Rzymski P., Perek B. (2017). Platelets redox balance assessment: current evidence and methodological considerations. *Vascular Pharmacology*.

[27] Chuang C. C., Shiesh S. C., Chi C. H. (2006). Serum total antioxidant capacity reflects severity of illness in patients with severe sepsis. *Critical Care*.

[28] Maciejczyk M., Szulimowska J., Skutnik A. (2018). Salivary biomarkers of oxidative stress in children with chronic kidney disease. *Journal of Clinical Medicine*.

[29] Poniedziałek B., Rzymski P., Pięt M. (2018). Relation between polyphenols, malondialdehyde, antioxidant capacity, lactate dehydrogenase and toxic elements in human colostrum milk. *Chemosphere*.

[30] Ayala A., Muñoz M. F., Argüelles S. (2014). Lipid Peroxidation: Production, Metabolism, and Signaling Mechanisms of Malondialdehyde and 4-Hydroxy-2-Nonenal. *Oxidative Medicine and Cellular Longevity*.

[31] Kambayashi Y., Binh N. T., Asakura H. W. (2009). Efficient assay for total antioxidant capacity in human plasma using a 96-well microplate. *Journal of Clinical Biochemistry and Nutrition*.

[32] Lubrano V., Balzan S. (2015). Enzymatic antioxidant system in vascular inflammation and coronary artery disease. *World Journal of Experimental Medicine*.

[33] Rice-Evans C., Miller N. J. (1994). Total antioxidant status in plasma and body fluids. *Methods in Enzymology*.

[34] Cracowski J. L., Cracowski C., Bessard G. (2001). Increased lipid peroxidation in patients with pulmonary hypertension. *American Journal of Respiratory and Critical Care Medicine*.

[35] Robbins I. M., Morrow J. D., Christman B. W. (2005). Oxidant stress but not thromboxane decreases with epoprostenol therapy. *Free Radical Biology & Medicine*.

[36] Fan Y. F., Zhang R., Jiang X. (2013). The phosphodiesterase-5 inhibitor vardenafil reduces oxidative stress while reversing pulmonary arterial hypertension. *Cardiovascular Research*.

[37] Bowers R., Cool C., Murphy R. C. (2004). Oxidative stress in severe pulmonary hypertension. *American Journal of Respiratory and Critical Care Medicine*.

[38] Smukowska-Gorynia A., Marcinkowska J., Chmara E. (2018). Neopterin as a biomarker in patients with pulmonary arterial hypertension and chronic thromboembolic pulmonary hypertension. *Respiration*.

[39] Masri F. A., Comhair S. A. A., Dostanic-Larson I. (2008). Deficiency of lung antioxidants in idiopathic pulmonary arterial hypertension. *Clinical and Translational Science*.

[40] Fessel J. P., Flynn C. R., Robinson L. J. (2013). Hyperoxia synergizes with mutant bone morphogenic protein receptor 2 to cause metabolic stress, oxidant injury, and pulmonary hypertension. *American Journal of Respiratory Cell and Molecular Biology*.

[41] Frijhoff J., Winyard P. G., Zarkovic N. (2015). Clinical relevance of biomarkers of oxidative stress. *Antioxidants & Redox Signaling*.

[42] Kaneda K., Fujita M., Yamashita S. (2010). Prognostic value of biochemical markers of brain damage and oxidative stress in post-surgical aneurysmal subarachnoid hemorrhage patients. *Brain Research Bulletin*.

[43] Wang Z., Yu C., Li X. H., Deng B. Q. (2017). The prognostic value of oxidative stress and inflammation in Chinese hemodialysis patients. *Renal Failure*.

[44] Bigagli E., Lodovici M. (2019). Circulating oxidative stress biomarkers in clinical studies on type 2 diabetes and its complications. *Oxidative Medicine and Cellular Longevity*.

[45] D'Arena G., Vitale C., Perbellini O. (2017). Prognostic relevance of oxidative stress measurement in chronic lymphocytic leukaemia. *European Journal of Haematology*.

[46] Sugimoto K., Sakamoto K., Kawai M. (2019). Serum oxidative stress is an independent prognostic marker in colorectal cancer. *Translational Cancer Research*.

[47] Ho E., Karimi Galougahi K., Liu C. C., Bhindi R., Figtree G. A. (2013). Biological markers of oxidative stress: Applications to cardiovascular research and practice. *Redox Biology*.

[48] DeMarco V. G., Habibi J., Whaley-Connell A. T. (2008). Oxidative stress contributes to pulmonary hypertension in the transgenic (mRen2) 27 rat. *American Journal of Physiology-Heart and Circulatory Physiology*.

[49] Fındıklı E., Camkurt M. A., İzci F. (2018). The diagnostic value of malondialdehyde, superoxide dismutase and catalase activity in drug naïve, first episode, non-smoker generalized anxiety disorder patients. *Clinical Psychopharmacology and Neuroscience*.

[50] Kono Y., Fridovich I. (1982). Superoxide radical inhibits catalase. *The Journal of Biological Chemistry*.

[51] Poniedziałek B., Rzymski P., Karczewski J. (2015). The role of the enzymatic antioxidant system in cylindrospermopsin-induced toxicity in human lymphocytes. *Toxicology In Vitro*.

[52] Lorente L., Rodriguez S., Sanz P. (2016). Association between pre-transplant serum malondialdehyde levels and survival one year after liver transplantation for hepatocellular carcinoma. *International Journal of Molecular Sciences*.

[53] Rusu C. C., Racasan S., Kacso I. M. (2016). Malondialdehyde can predict survival in hemodialysis patients. *Medicine and Pharmacy Reports*.

[54] Young I. S. (2001). Measurement of total antioxidant capacity. *Journal of Clinical Pathology*.

[55] Pérez-Torres I., Guarner-Lans V., Rubio-Ruiz M. E. (2017). Reductive stress in inflammation-associated diseases and the pro-oxidant effect of antioxidant agents. *International Journal of Molecular Sciences*.

[56] de Wijs-Meijler D. P., Duncker D. J., Tibboel D. (2017). Oxidative injury of the pulmonary circulation in the perinatal period: short- and long-term consequences for the human cardiopulmonary system. *Pulmonary Circulation*.

[57] Gryszczyńska B., Formanowicz D., Budzyń M. (2017). Advanced oxidation protein products and carbonylated proteins as biomarkers of oxidative stress in selected atherosclerosis-mediated diseases. *BioMed Research International*.

